# Continuous decoding of movement intention of upper limb self-initiated analytic movements from pre-movement EEG correlates

**DOI:** 10.1186/1743-0003-11-153

**Published:** 2014-11-15

**Authors:** Eduardo López-Larraz, Luis Montesano, Ángel Gil-Agudo, Javier Minguez

**Affiliations:** DIIS, Universidad de Zaragoza, María de Luna, 1, Zaragoza, Spain; Instituto de Investigación en Ingeniería de Aragón, Zaragoza, Spain; Unidad de Biomecánica y Ayudas Técnicas, Hospital Nacional de Parapléjicos, Toledo, Spain; Bit & Brain Technologies SL, Zaragoza, Spain

**Keywords:** Electroencephalography, Brain-machine interfaces, Event-related desynchronization, Motor-related cortical potentials, Analytic movements, Motor rehabilitation, Spinal cord injury, Automatic feature selection

## Abstract

**Background:**

Brain-machine interfaces (BMI) have recently been integrated within motor rehabilitation therapies by actively involving the central nervous system (CNS) within the exercises. For instance, the online decoding of intention of motion of a limb from pre-movement EEG correlates is being used to convert passive rehabilitation strategies into active ones mediated by robotics. As early stages of upper limb motor rehabilitation usually focus on analytic single-joint mobilizations, this paper investigates the feasibility of building BMI decoders for these specific types of movements.

**Methods:**

Two different experiments were performed within this study. For the first one, six healthy subjects performed seven self-initiated upper-limb analytic movements, involving from proximal to distal articulations. For the second experiment, three spinal cord injury patients performed two of the previously studied movements with their healthy elbow and paralyzed wrist. In both cases EEG neural correlates such as the event-related desynchronization (ERD) and movement related cortical potentials (MRCP) were analyzed, as well as the accuracies of continuous decoders built using the pre-movement features of these correlates (i.e., the intention of motion was decoded before movement onset).

**Results:**

The studied movements could be decoded in both healthy subjects and patients. For healthy subjects there were significant differences in the EEG correlates and decoding accuracies, dependent on the moving joint. Percentages of correctly anticipated trials ranged from 75*%* to 40*%* (with chance level being around 20*%*), with better performances for proximal than for distal movements. For the movements studied for the SCI patients the accuracies were similar to the ones of the healthy subjects.

**Conclusions:**

This paper shows how it is possible to build continuous decoders to detect movement intention from EEG correlates for seven different upper-limb analytic movements. Furthermore we report differences in accuracies among movements, which might have an impact on the design of the rehabilitation technologies that will integrate this new type of information. The applicability of the decoders was shown in a clinical population, with similar performances between healthy subjects and patients.

**Electronic supplementary material:**

The online version of this article (doi:10.1186/1743-0003-11-153) contains supplementary material, which is available to authorized users.

## Introduction

Motor rehabilitation with robotic or functional electrical stimulation exercises has emerged as a promising alternative to design new therapies for stroke or spinal cord injury (SCI) patients [1–3]. An emerging trend in these new therapies is to shift from passive mobilizations to exercises that involve the central nervous system (CNS) in an active way [4], as it has been shown that this type of rehabilitation enhances neuroplasticity [5, 6]. Movement intention is a key state that expresses the CNS active involvement with well-established EEG correlates (e.g., the event-related desynchronization/synchronization, ERD/S [7], or the motor-related cortical potentials, MRCP [8]). These correlates have the advantage of anticipating any peripheral measurement, which make them suitable to build more usable and natural BMIs [9, 10], and allow high temporal precision for the control of prosthetic or orthotic devices [11]. In addition, they are measurable even in paralyzed patients [12–15]. During early stages of rehabilitation (e.g., in acute and subacute phases after a stroke or SCI), physiotherapy exercises include analytic movements (i.e., mobilization of single joints), as they help to improve joint range of motion and muscular activity, enhance oxygen metabolism, and can be performed before the patients are ready to execute more complex movements [16, 17]. To evaluate the applicability of technologies that use the movement intention in the early stages of rehabilitation, there is a need to understand how this intention can be decoded under a wide range of analytic movements. As a first step, this paper analyzes the possibility of building decoders for seven upper-limb analytic movements using pre-movement EEG correlates with healthy subjects. In addition, the proposed decoders are tested in a clinical environment with a small cohort of SCI patients.

EEG continuous decoding of motor intention has been achieved using either ERD/S or MRCP correlates for functional movements such as reaching tasks [12, 14]; for multiple joint movements such as simultaneous movement of both feet [18] or hand movement imagination [13, 19]; and for single joint analytic movements such as wrist extension [10, 20, 21] or ankle dorsiflexion [22, 23]. Statistical differences in ERD/S and MRCP correlates have been demonstrated not only between rest and motion (or motor imagery) but also between different types of movements [8, 24–26], which is why they have been used to distinguish between different motor tasks [9, 27–29]. Since they reflect different neurophysiological phenomena [30], their combination has been proven to outperform single trial classification of movements [31, 32], and decoding of motion intention [33].

This paper shows the applicability of BMIs in upper-limb self-initiated analytic movements with two different experiments. The first experiment studies the seven degrees of freedom of the arm. This experiment was performed on healthy subjects so that they could perform all the movements correctly. The second experiment evaluates, on a target population (SCI patients), the applicability of the BMI with two of the previous movements: one that they could perform normally, and a second one that they could not execute completely. ERD and MRCPs of the different movements were studied in both healthy and SCI patients. The decoders of movement intention (pre-movement state) combined ERD and MRCP features using a sparse feature selection method. The results shed light on: *(i)* the differences of the neural correlates on an ample set of upper-limb movements, *(ii)* the applicability of BMI technology to the different arm movements on healthy subjects as well as SCI patients, and *(iii)* on practical issues such as the automation of the feature selection process and decoding time-anticipation (which is important for incorporating feedback strategies that trigger neuroplasticity mechanisms [11]).

## Methods

### Experimental protocols

Two different experiments were designed for this study. The first one shows the differences in BMI applicability for the seven degrees of freedom of the arm on healthy subjects. The second experiment validates the use of the proposed BMI on a clinical population of SCI patients. All participants (healthy subjects and patients) were duly informed before the experiment and all of them provided written informed consent. The experimental procedure was approved by the Ethics Review Board of the *Hospital Nacional de Parapléjicos* (Toledo, Spain).

#### Experiment 1

**Figure 1:**
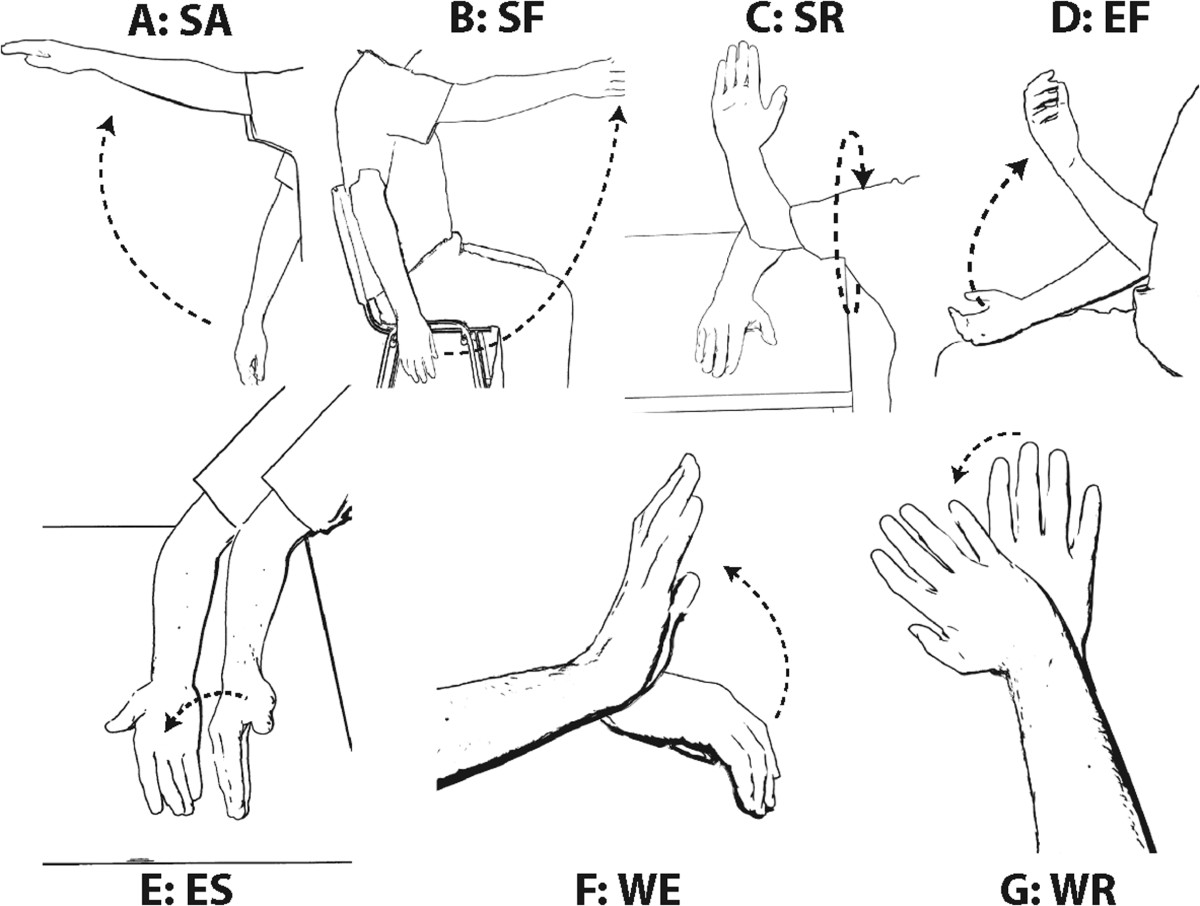
**Representation of the seven upper limb analytic movements performed by the subjects.** Panels **A-G** correspond to each of the seven movements (SA, SF, SR, EF, ES, WE, and WR, respectively) performed by the subjects. The arrows indicate the direction of the movement.

Six healthy right-handed male subjects (mean age 26.66±2.94 years) participated in this experiment. Participants were comfortably seated 1.5 m away from a computer screen, which displayed the instructions during the experiment. The chair incorporated a removable armrest that was only used for movements that required it. Additionally, a supplementary table was used to hold the arm in an elevated rest position for movements that required that support. Seven analytic movements of the right upper limb were studied (Figure 1). They were selected to evaluate the seven degrees of freedom of the three arm joints separately. As each movement had a different initial position, the participants started each block with the arm in the position required for the upcoming task:

SA: Shoulder abduction-adduction (Figure 1A). Initial position: arm hanging straight down.

  ● Task: 90° abduction (separation) and adduction (approximation) to initial position.

SF: Shoulder flexion-extension (Figure 1B).

  ● Initial position: arm hanging straight down.

  ● Task: 90° flexion (forward) and extension (backwards) to initial position.

SR: Shoulder external-internal rotation (Figure 1C).

  ● Initial position: elbow resting on a table situated next to subject.

  ● Task: 90° external rotation (upwards) and return to initial position.

EF: Elbow flexion-extension (Figure 1D).

  ● Initial position: arm resting over subject’s leg, palm upwards.

  ● Task: maximal elbow flexion and extension to initial position.

EF: Elbow supination-pronation (Figure 1E).

  ● Initial position: elbow resting over the chair armrest, hand open and parallel to the wall.

  ● Task: 90° supination (palm upwards) followed by 90° pronation (palm downwards) and return to initial position.

WE: Wrist extension-flexion (Figure 1F).

  ● Initial position: elbow lying over the chair armrest, hand open and parallel to the floor, palm downwards.

  ● Task: maximal wrist extension (upwards) and flexion to initial position.

WR: Wrist radial-ulnar deviation (Figure 1G).

  ● nitial position: elbow lying over the chair armrest, hand open and parallel to the floor, palm downwards.

  ● Task: radial deviation (inner movement) followed ulnar deviation (outer movement) and return to initial position.

Experimental sessions consisted of 14 blocks of trials (2 blocks per movement) in the following order: SA-SF-SR-EF-ES-WE-WR-SA-SF-SR-EF-ES-WE-WR. After each block, participants could rest as much time as they required. Each block included 25 trials of one movement type (totaling 50 recorded trials per movement). Trials lasted 13 seconds (block duration = 5´25˘); the screen showed the word *Movement* during the first 10 seconds and the word *Rest* during the last 3 seconds. Subjects were instructed to perform the analytic movement whenever they wanted during the *Movement* cue (self-initiated action), waiting at least 3 seconds after the cue appeared. They were explicitly asked to avoid mentally counting before starting movement. The *Rest* screen indicated to the subjects that they could blink and relax.

#### Experiment 2

Three male incomplete quadriplegic patients were recruited for this experiment. All of them were in a subacute phase and hospitalized at the *Hospital Nacional de Parapléjicos*, in Toledo (Spain), where the experimentation sessions took place. As inclusion criteria, patients’ right arm should have the elbow flexors intact (score 5 out of 5 in a muscle strength scale [34]), and should have weakness in the wrist extensors (muscle strength 2-3 out of 5). Relevant information of each patient can be found on Table 1. This typology of lesion was chosen as it allowed to study EEG correlates and decoding performances of an arm movement that the patients can perform normally and a movement that they cannot execute despite them still having some muscle strength. In addition, these patients are more autonomous than the ones with higher and complete lesions (who frequently have breathing difficulties) and thus are better able to participate in a BMI intervention setup.

**Table 1:** **Details of patients**

	Age	Time since	Type of	Muscle strength	Muscle strength
ID	(years)	lesion (months)	lesion	elbow flexors	wrist extensors
P1	36	7	C7, ASIA B	5	3
P2	38	10	C5, ASIA C	5	2
P3	55	4	C5, ASIA D	5	2

During the experiment, patients were seated in their wheelchair 1.5 meters away from a computer screen, which displayed the instructions. They were asked to perform two different analytic movements: EF (complete movement as healthy participants) and WE (to the maximum extension they were able to perform). Patients executed 6 blocks of 20 trials each (3 blocks per movement), totaling 60 trials of each movement. Blocks of both types of movements were intercalated, and patients could rest as long as they required after each block. The structure of these trials was the same as in Experiment 1. Patients were also instructed to perform self initiated movements and to avoid mentally counting.

### Data acquisition

EEG, EMG, and inertial measurement unit (IMU) signals were acquired in Experiment 1, while only EEG and EMG were recorded for Experiment 2 (as it was not expected to have a complete movement in one of the tasks). EEG and EMG signals were recorded together using a commercial g.Tec system (g.Tec GmbH, Graz, Austria). EEG configuration consisted of 32 active electrodes placed at AFz, F3, F1, Fz, F2, F4, FC5, FC3, FC1, FCz, FC2, FC4, FC6, C5, C3, C1, Cz, C2, C4, C6, CP5, CP3, CP1, CPz, CP2, CP4, CP6, P3, P1, Pz and P4 (according to the international 10/10 system). The ground and reference electrodes were placed on FPz and on the left earlobe, respectively. EMG setup consisted of 8 bipolar electrodes, placed at the right arm on top of the following muscles: *(i)* extensor digitorum, *(ii)* extensor carpi ulnaris, *(iii)* palmaris longus, *(iv)* external head of the biceps brachii, *(v)* lateral head of the triceps brachii, *(vi)* frontal side of the deltoid, *(vii)* lateral side of the deltoid, and *(vii)* posterior side of the deltoid over the teres minor and infraspinatus muscles. EEG and EMG signals were digitized at a sampling frequency of 512 Hz and power-line notch-filtered to remove the 50Hz line interference. An IMU acquisition system (Technaid S.L., Madrid, Spain) recorded accelerometer, gyroscope and magnetometer signals at a sampling frequency of 50 Hz. Sensors were placed over the three arm segments (hand, forearm, and arm), and over the trunk (sternum). After Experiment 1, the EEG, EMG and IMU signals were synchronized by using an artificial external pulse that was generated by a computer and sent to both recording systems.

### Signal preprocessing

For Experiment 1, IMU signals were resampled to 512 Hz using interpolation to match the EEG and EMG signals, and the three measurements were aligned using the artificial pulse. Information from the EMG and IMU sensors was evaluated to identify the onset of movements. However, since EMG signals were corrupted for certain subjects, IMU channels with the highest amplitudes and best consistency with the EMG activations were manually selected for each movement and subject in order to obtain the movement onsets. The movement onset detection procedure was as follows: (*i*) the mean of the selected IMU channels was removed, and the signals were rectified; (*ii*) when more than one IMU channel were selected, their values were averaged; (*iii*) the movement onsets were defined as the time instants in which the signal was at 5% of the maximum amplitude. For Experiment 2, EMG signals of the biceps muscle and of extensor digitorum muscle were selected to detect movement onset of EF and WE, respectively, following the procedure used in [22].

EEG signals were trimmed down to 6-second trials (-3 to 3 seconds from the movement onset). Trials with movements that started before the 3-second waiting period were discarded. For Experiment 1, EEG channels FC3 and FC1 were removed from all the subjects, as they presented artifacts for all movements. Additionally, a z-score artifact rejection (similar to the one proposed in [35]) was performed on the following computed measurements: the power in *δ* (1-4 Hz), *θ* (4-7 Hz), *α* (7-12 Hz), and *β* (12-30 Hz) frequency bands, the trial variance and the maximum amplitude, discarding trials that contained values higher than 2.5 times the mean.

### Optimal spatial filtering

Spatial filters such as CAR, Laplacian, common spatial patterns (CSP) and methods based on optimization techniques are broadly used to obtain reference-free signals and to enhance the signal to noise ratio (SNR) [22, 36, 37]. This paper evaluates the use of optimal spatial filters (OSF) to improve the SNR in ERD and MRCP signals. The OSF are computed for both signals (ERD and MRCP) as a linear combination of all EEG channels [22]. First, EEG trials were bandpass filtered to [0.1-1] Hz for MRCPs, and [7-30] Hz for ERD. Next, the trials were segmented in the time intervals [-1, 1] s to represent the signal of interest (denoted by *S*_*EEG*_) and [-3, -1] s for the noise (*N*_*EEG*_). Note that 0 is the time of the movement onset.

Given a set of *L* epochs of *S*_*EEG*_ and *L* epochs of *N*_*EEG*_, the SNR was computed as:

$$\text{SNR}=\frac{1}{L}\overset{L}{\underset{i=1}{\sum }}10\;\text{lo}g_{10}\left(\frac{P_{\text{Si}}}{P_{\text{Ni}}}\right)$$

where *P*_*Si*_ and *P*_*Ni*_ represent the power of the signal of interest ($S_{i}=w\cdot S_{\text{EE}G_{i}}$) and noise ($N_{i}=w\cdot N_{\text{EE}G_{i}}$) for the *i*th trial, respectively. The vector of coefficients *w*=(*w*_1_,…,*w*_*c*_) contained the weights for each channel, and it included 30 values for signals of Experiment 1 (since 2 channels were removed, see Signal preprocessing section) and 32 for Experiment 2.

This procedure was performed separately for MRCP and ERD (i.e., the application of the OSFs generated two new signals, denoted hereafter as *OSF signals*: one to optimize ERD and the other to optimize MRCP). For MRCP the SNR was maximized, as there is a need to have high amplitude in the interval of interest, and low amplitude in the noise period [22]. In contrast, for ERD the SNR was minimized, as there is a decrease in power during movement with respect to the non-movement or noise interval [38]. A constrained optimization solver was used (Matlab function fmincon), setting the sum of *w* to be equal to zero. The vector *w* was initialized as a CAR filter for channel Cz when computing the OSF of MRCP, and as a CAR filter for channel C3 in the case of ERD.

### Electrophysiology analysis

ERD and MRCP were analyzed for each movement on the EEG channels and the optimized OSF signals. The event-related desynchronization analysis was used to measure the power modulations in *α* and *β* frequency bands. The EEG trials were filtered using small Laplacian derivations to reduce the effects of volume conduction [39]. Next, the filtered channels and the OSF signal were bandpass filtered between 1 and 50 Hz using a zero-phase fourth-order Butterworth filter. The time-frequency representation of each subject and movement was computed using Morlet Wavelets in the frequency range [1-50] Hz [40]. Statistical significance (*α*=0.05) of the time-frequency analysis was computed with respect to baseline [-3, -1] using a bootstrap resampling method [38]. To compute the motor-related cortical potentials, EEG trials were filtered with a common average reference (CAR). Filtered trials and OSF signal were subsampled to 64 Hz, and bandpass filtered to the frequency range [0.1-1] Hz [41]. A zero-phase second-order Butterworth filter was used, as it worked just as well as the finite impulse response filter suggested in [41], and in a lower order.

Movements were grouped according to their joint (i.e., shoulder, elbow and wrist) by averaging the values of the movements corresponding to each of them. ERD and MRCP differences between joints were studied using one-way repeated measures analysis of variance (ANOVA) with the within-subjects factor moving joint. Post-hoc comparisons were done using paired t-tests with Bonferroni correction.

### Feature extraction and classification

For each time instant *t* the following features were computed using a one-second time window [*t*−1,*t*]:

**ERD:** A Laplacian filter was applied to the EEG channels and the power spectra was computed using a 16*th* order autoregressive model with a frequency resolution of 1 Hz [42]. Power values in all frequency bins that included *α* and *β* bands were selected (7-30 Hz). The 19 channels comprising fronto-central (FCx), central (Cx), and centro-parietal (CPx) areas were chosen. In total, 456 features were generated. Additionally, the same steps were applied to compute the power spectra of the OSF signal, which produced 24 features.

**MRCPs:** The EEG trials were CAR filtered, subsampled to 64 Hz and filtered in the range [0.1-1] Hz. Time samples in channels FCz, FC2, C1, Cz, C2, CP1, CPz, CP2 were selected. In total 512 features were computed. For the OSF signal the application of the same steps generated 64 extra features.

**Figure 2:**
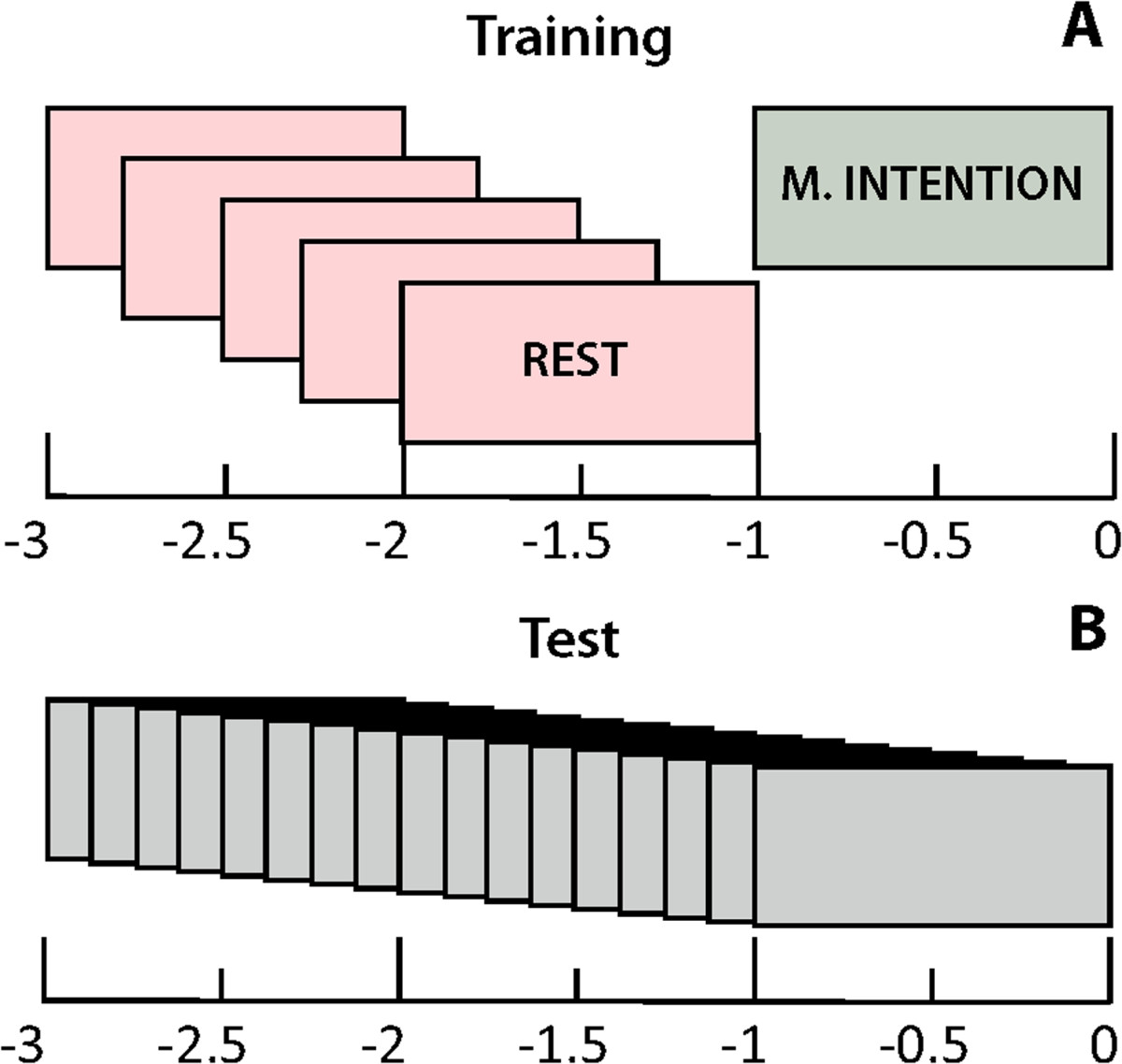
**Schematic view of the training and test operation of the system.** **(A)** Training procedure extracted five time windows to model rest process and one to model movement intention. **(B)** Test operation of the system worked with a sliding window that moved along the trial with a sliding step of 0.125 s.

Therefore, the feature vectors of each time window were composed of 1056 values. Dimension reduction of feature vectors is usually performed automatically with separability metrics like Bhattacharyya or Mahalanobis distances [10, 28]. However, these methods can sometimes provide highly correlated features in EEG measurements. Therefore, in this paper we used an automatic selection procedure based on sparsity to select the most discriminant values from a large set of features, while removing redundant information [43]. Sparse discriminant analysis (SDA) performs linear discriminant analysis (LDA) with a sparseness criterion imposed such that the feature selection and classification are performed simultaneously [43]. This allows the estimation of covariance matrices in datasets with the number of features being large relative to the number of observations, as it was in our case. Feature vectors corresponding to the training set were processed with this algorithm, which was programed to select, from each vector, a number of features always inferior to the number of trials available for training. When computing the OSF for both ERD and MRCP features, coefficients were estimated using only the training set, and these coefficients were then applied to the test set.The classifier was trained to distinguish between the rest state and the pre-movement state from the brain signals. The examples of the rest class were the one-second-long time windows in the time interval [-3, -1] s computed with a sliding step of 0.25 s, and the movement intention was the window [-1, 0] s (Figure 2A). Note that no information of the actual movement was considered for training, as the objective of the BMI was to detect the movement before it occurred. Feature vectors were obtained from each time window in the training dataset, and their values were normalized to have zero mean and unit variance before training the classifier. Trials of the test set were evaluated with a sliding window applied to the time interval [-3, 0], with a sliding step of 0.125 s (Figure 2B).

### Classifier evaluation and metrics

Classification of the trials from each subject and movement was evaluated separately. Given the reduced number of trials for each condition (after artifacting *N*≤50 for Experiment 1 and *N*≤60 for Experiment 2), the classification performance was evaluated using a trial-based leave-one-out cross-validation. For each fold, the classifier was trained using *N*−1 trials, and the test trial was evaluated with a sliding window.

Three different metrics were used to evaluate the classifier performance, which were computed on a trial basis, instead of using a sample-by-sample analysis that evaluates each test window independently. Firstly, ROC curves were used to evaluate the sensitivity and specificity [44], and the area under the curve (AUC) was used as performance metric, since it is threshold independent and also invariant to *a priori* probabilities [45]. Furthermore, it has been proposed to use an event-by-event analysis to better model the behavior of a continuous decoder with ROC curves [19]. Sensitivity and specificity were definied considering rest and movement intention periods as events. Thus, true positive events (TPE) were defined as correct onset detections made in the time interval [-1, 0]; false positive events (FPE) as events with at least one classifier onset misclassification during the rest phase [-3, -1]; true negative events (TNE) as rest periods with no false onset detections; and false negative events (FNE) as movement intention periods with no correct onset detections. Secondly, the percentage of correct trials was measured. This metric captures the number of executions that would provide a correct detection (i.e., a correct trigger for the robot or electrical stimulator during a therapy) before any other peripheral measurement [10]. A trial was correct if no intention of movement was detected before *t*=−1 s, and if an intention was detected between *t*=−1 s and *t*=0 s with respect to the actual onset (i.e., its rest period was a TNE, and its movement intention was a TPE). Thirdly, for all correct trials, their anticipation was calculated as the period between the detection time and the movement onset. Given the definition of correct trials, only activations in the period [-1, 0] s were taken into account.

## Results

### Experiment 1

#### Electrophysiology analysis

**Figure 3:**
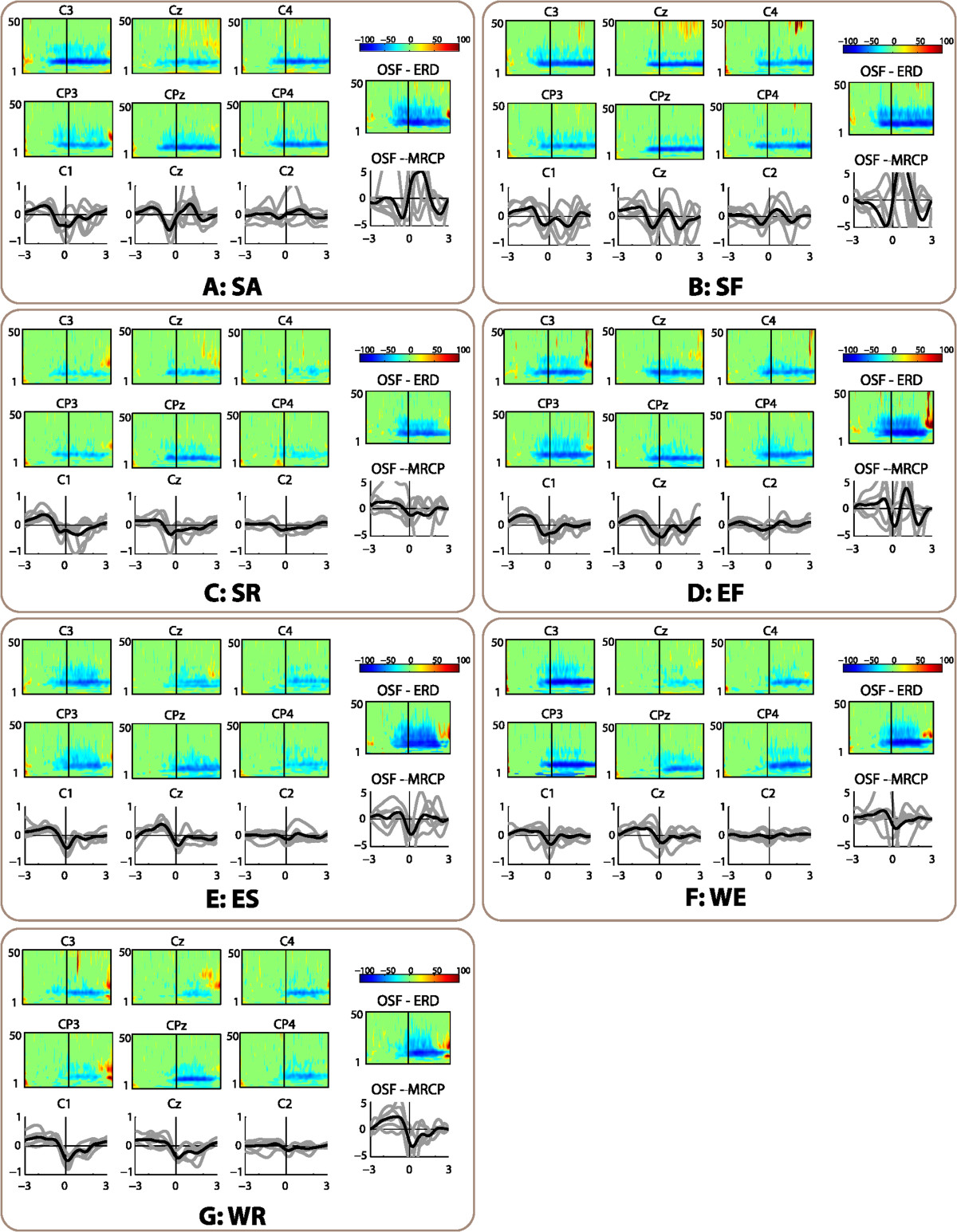
**Electrophysiology analysis of the seven movements, averaged for the six healthy subjects.** Panels **A-G** correspond to the analysis for each of the seven movements (SA, SF, SR, EF, ES, WE, and WR, respectively). The top and center-left areas of each panel show the significant ERD process in six channels around the motor cortex (*x* axis corresponds to the time interval [-3, 3], *y* axis represents the frequency range [1-50] Hz). Bottom-left area shows the average MRCPs for each subject (gray lines), and the average of the six subjects (black lines) in central channels C1, Cz and C2. Top-right depicts the scale of ERD plots. Center-right shows the ERD in the OSF signal. Bottom-right corresponds to the MRCP of the OSF signal.

Significant ERD activity (*p*<0.05) in *α* (7-12 Hz) and *β* (12-30 Hz) frequency bands was found more prominently over the contralateral motor cortex before and during the movement for almost all movements and subjects. Figure 3 displays the ERD averaged for all subjects over the motor cortex for each movement. Additionally, Table 2 synthesizes the pre-movement (time interval [-1, 0] s) ERD values of channel C3 for each subject and movement, and the averages for movements and subjects. Notice that averaged pre-movement ERD was higher for proximal than for distal movements. ANOVA showed statistical significance over the pre-movement *α* ERD (*F*_2,10_=5.7,*p*<0.05) but not on the *β* ERD. Post-hoc pair-wise tests with Bonferroni correction revealed differences between the shoulder and the wrist (*p*=0.008).

**Table 2:** **Pre-movement** ***α*** **and** ***β*** **ERD** ***%*** **- channel C3**

		SA	SF	SR	EF	ES	WE	WR	Avg
S1	*α*	-76.9	-74.5	-28.3	-76.3	-68.5	-54.4	-3.3	-54.6
	*β*	-49.1	-48.6	-17.1	-47.5	-36.8	-19.8	-6.5	-32.2
S2	*α*	-35.1	-27.5	-2.9	-25.2	-29.0	-10.9	-0.6	-18.7
	*β*	-24.6	-12.8	-6.5	-25.9	-17.6	-4.3	-3.2	-13.6
S3	*α*	-72.5	-63.4	-41.4	-43.5	-47.4	-36.1	-26.5	-47.3
	*β*	-41.5	-31.2	-2.6	-10.3	-11.9	-20.6	-6.7	-17.8
S4	*α*	-17.3	-1.3	-6.4	-24.0	0.0	-1.4	-1.0	-7.3
	*β*	0.0	0.0	-3.0	-3.7	-0.9	-6.5	0.0	-2.0
S5	*α*	-38.7	-39.7	-20.5	-19.9	-22.0	-7.1	-2.9	-21.6
	*β*	-34.1	-34.1	-10.3	-11.1	-28.9	-7.7	-17.9	-20.6
S6	*α*	-41.7	-23.4	-60.8	-9.6	-7.4	-36.7	-13.5	-27.6
	*β*	-1.6	-14.6	-10.4	-3.0	-0.9	-12.9	-4.3	-6.8
**Avg**	*α*	-47.0	-38.3	-26.7	-33.1	-29.1	-24.4	-8.0	-29.5
	*β*	-25.2	-23.5	-8.3	-16.9	-16.2	-12.0	-6.4	-17.0

Averaged MRCPs for all subjects and for each type of movement are displayed in Figure 3. The values of all the MRCPs were normalized to the peak value of the movement with highest amplitude for each subject. The lateralized cortical potential towards contralateral motor area was present for all movements and subjects. Table 3 summarizes the normalized amplitudes of the MRCP negative peaks of channels Cz and C1. There were no statistical differences between joints in peak amplitude for channels Cz (*p*>0.05) and C1 (*p*>0.05).

**Table 3:** **MRCPs min peak normalized amplitude**

		SA	SF	SR	EF	ES	WE	WR	Avg
S1	Cz	-0.47	0.00	-0.66	-0.69	-0.46	-0.28	-0.47	-0.43
	C1	-0.58	-0.71	-1.00	-0.63	-0.74	-0.02	-0.35	-0.58
S2	Cz	-0.23	-0.30	-0.17	-0.27	-0.42	-0.43	-0.47	-0.33
	C1	-0.31	-1.00	-0.32	-0.47	-0.63	-0.80	-0.83	-0.62
S3	Cz	-0.35	-0.61	-0.24	-0.68	-0.18	-0.67	-0.41	-0.45
	C1	-1.00	-0.76	-0.26	-0.40	-0.12	-0.41	-0.38	-0.48
S4	Cz	-0.76	-0.64	-1.00	-0.68	-0.03	-0.76	-0.53	-0.63
	C1	-0.60	-0.48	-0.64	-0.62	-0.46	-0.40	-0.73	-0.56
S5	Cz	-1.00	-0.34	-0.46	-0.51	-0.61	-0.18	-0.59	-0.52
	C1	-0.79	-0.46	-0.31	-0.38	-0.45	-0.42	-0.59	-0.48
S6	Cz	-0.53	-1.00	-0.31	-0.40	-0.41	-0.40	-0.16	-0.46
	C1	-0.41	-0.76	-0.31	-0.30	-0.35	-0.43	-0.30	-0.41
**Avg**	Cz	-0.56	-0.48	-0.47	-0.54	-0.35	-0.45	-0.44	-0.47
	C1	-0.62	-0.69	-0.47	-0.47	-0.46	-0.41	-0.53	-0.52

The ERD in the OSF signal (right area of each panel in Figure 3) was significantly higher than in C3 for *α* band (Wilcoxon matched pairs signed-rank test, *p* < 10^−7^) and *β* band (*p* < 10^−7^). Averaged ERD for all subjects and movements in channel C3 was −29.5*%* in *α* band and −17.0*%* in *β* band (bottom-right values in Table 2), while in the OSF signal were −54.9*%* and −36.6*%*, respectively. The amplitude of MRCPs was also significantly higher in the OSF signal than in Cz (*p* < 0.001) and C1 (*p* < 0.01). While the average normalized amplitude was −0.47 in Cz and −0.52 in C1 (Table 3), the OSF signal had an average amplitude of −2.94 in the same normalized scale.

#### Classification results

**Figure 4:**
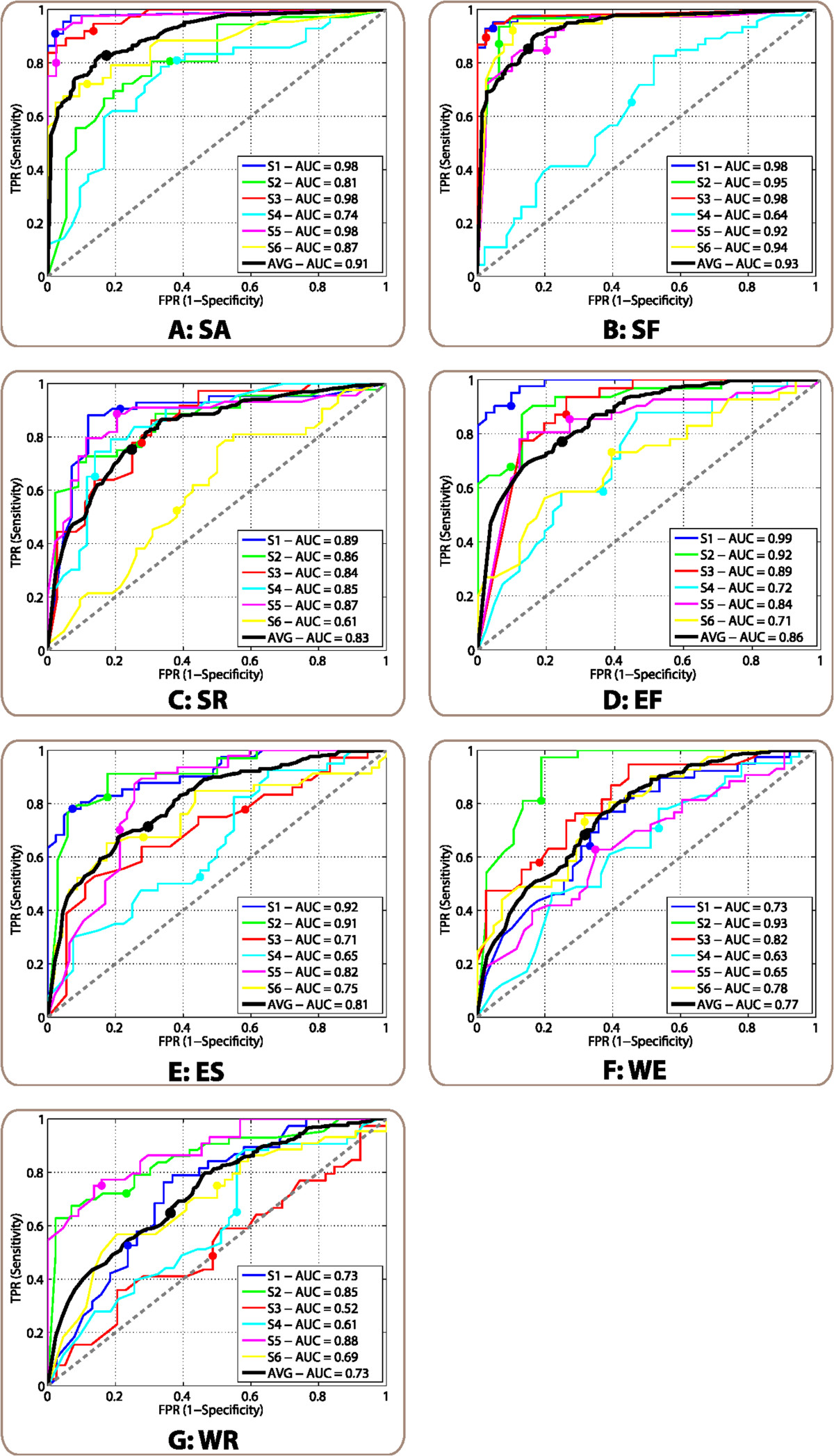
**ROC curves obtained for each movement.** Panels **A-G** correspond to the results for each of the seven movements (SA, SF, SR, EF, ES, WE, and WR, respectively). On each panel, colored lines represent the different subjects, while the black line shows the average of the six subjects. Circles on each line represent the point of the curve with equal sensitivity and specificity, which are considered the optimal working points. Diagonal gray dashed lines represent the performance of a random classifier. Each legend box contains the AUC for each subject and the average of them.

Average ROC curves for each movement are shown in Figure 4. Circles on each line represent the working points where sensitivity equals specificity, which give the optimal thresholds [19]. Proximal movements presented higher AUC than distal ones, in agreement with the electrophysiology analysis, which showed higher average values of ERD and MRCP peaks. With the optimal threshold in the ROC curve, the movement with highest AUC (SF) had 85.6*%* of true positives and 15.1*%* of false positives. On the other hand, for the movement with poorest AUC (WR), the decoder provided 64.8*%* of true positives and 36.2*%* of false positives. Pre-movement *α* and *β* ERD showed significant correlation with AUC (*r*_*α*_=−0.49, *p*_*α*_<10^−3^; *r*_*β*_=−0.66, *p*_*β*_<10^−5^). Regarding MRCP peak amplitude, it also showed a significant correlation for channels C1 (*r*_*C*1_=−0.57, *p*_*C*1_<10^−4^), and Cz (*r*_*Cz*_=−0.36, *p*_*Cz*_<0.02). ANOVA showed a significant effect of moving joint in AUC (*F*_2,10_=8.0,*p*<0.01). However, post-hoc comparisons after Bonferroni correction did not show significant differences, although differences between shoulder and wrist were close to the significance level (*p*=0.064).

Table 4 shows the percentage of correct trials for each movement averaged for all subjects. Movements with higher AUC provided better rates of accepted trials. In all cases, the number of correct trials was higher than the empirical chance level (obtained with the same leave-one-out procedure, but randomizing the labels before training the classifier), which provided on average 21.2±3.4*%* of correct trials. There was a significant effect on the moving joint in percentage of correct trials as revealed by ANOVA (*F*_2,10_=10.6,*p*<0.01), with significant differences between shoulder and wrist (*p*=0.04).

**Table 4:** **Percentage of correct trials averaged for healthy subjects**

	SA	SF	SR	EF	ES	WE	WR	Avg
Avg Healthy	72.1±17.7*%*	74.4±24.6*%*	57.4±19.3*%*	60.8±15.4*%*	53.2±16.3*%*	47.6±10.3*%*	39.5±17.6*%*	57.9±12.6*%*
Subjects								

**Figure 5:**
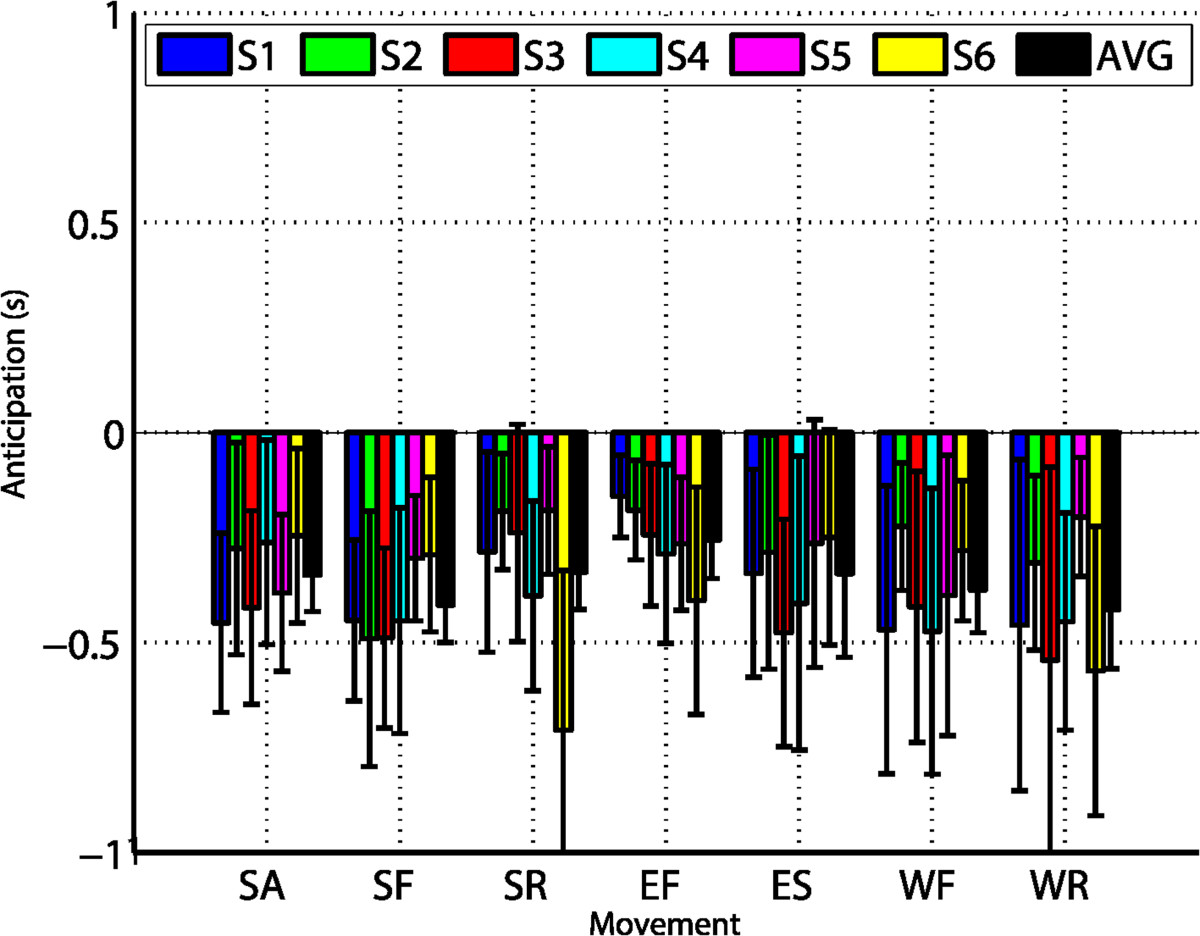
**Results of anticipation analysis.** The *y* axis indicates the anticipation in seconds and the *x* axis the movement type. For each movement, the colored bars indicate the mean ±std anticipation of each subject, and black bars the average anticipation for all of them.

Average onset detection times ranged from -421 ms in the best case (WR) to -256 ms for the least anticipated movement (EF), with respect to the movement onset (Figure 5). The moving joint had a significant effect in anticipation, shown by ANOVA (*F*_2,10_=8.6,*p*<0.01), and post-hoc comparisons revealed statistical differences between elbow and wrist movements (*p*=0.048).

#### Analysis of features

**Figure 6:**
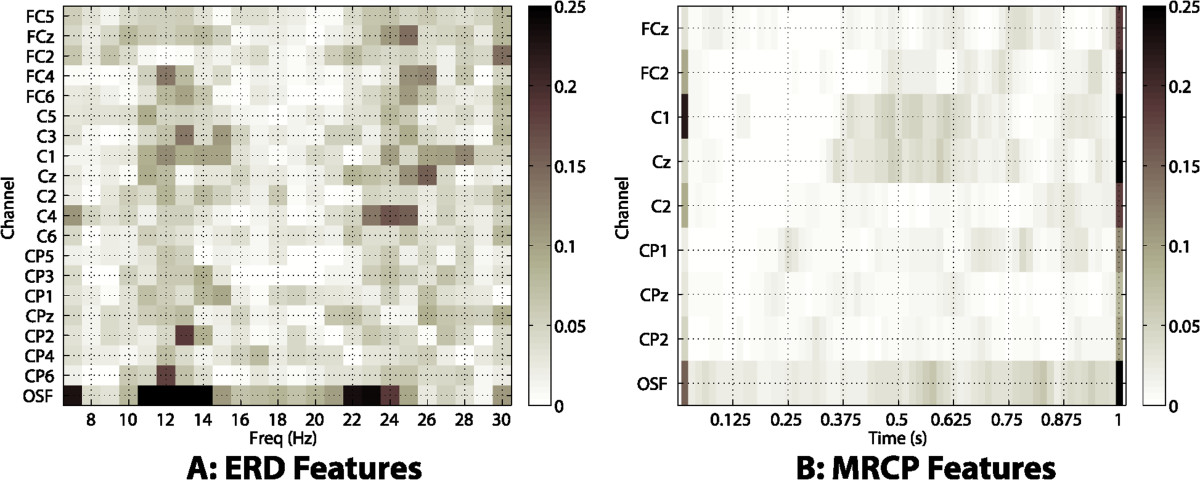
**Features selected by SDA.** For their representation, information of all subjects and movements was combined. In both cases the last row corresponds to the OSF signal. The scale of both figures represents the percentage of times that the features were chosen over all folds of all leave-one-out executions. **(A)** Channels and frequencies for ERD features. **(B)** Channels and time samples of MRCP features.

As features were selected automatically by the SDA, an analysis was performed to understand the information used by the algorithm. Figure 6 shows a projection of the features selected for each fold of the leave-one-out procedure to the channels-frequencies space (for ERD), and to the channels-time space (for MRCP), averaged for all subjects and movements. For ERD, bins in the frequencies 7 Hz (lower *α*), [10-15] Hz (upper *α*) and [22-25] Hz (*β*) were more often selected in the OSF channel, but also in channels placed over ipsi and contralateral motor cortices (e.g., C3, C1, C4). Regarding time features of MRCP, note how the most frequently selected were the last samples of the time-window (samples in the figure above *t*=1 s), which coincide with the maximum peak of the MRCP.

**Figure 7:**
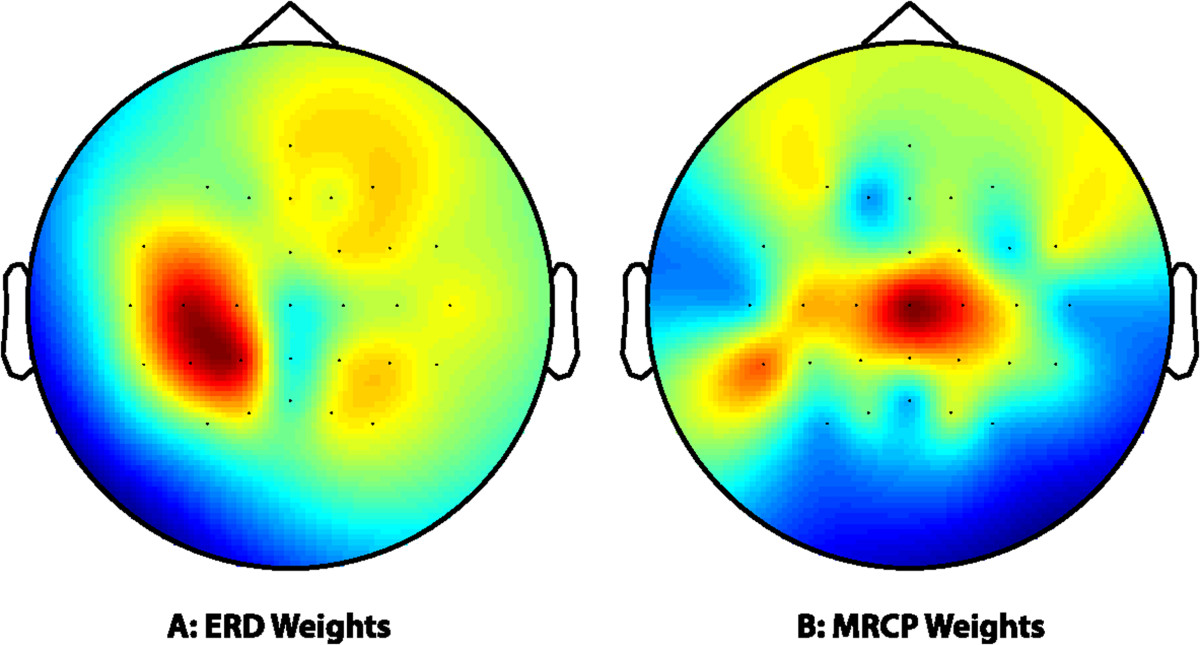
**Weights assigned by the optimal filters to each channel for ERD and MRCP.** The topoplots show the average weights for all subjects and movements given to each channel for ERD **(A)** and MRCP **(B)**. Red color represents higher, while blue represent lower weights.

The OSF signal was the most frequently selected in both cases. Thus the mean weight given to each channel for the OSF computed for each subject and movement was obtained. Figure 7 shows the weights obtained for ERD (Figure 7A) and MRCP (Figure 7B) filters, averaged for all subjects and movements. The figures show that the left motor cortex (contralateral to the movement) and the central area of the motor cortex were the areas with the highest weights to build the OSF for ERD and MRCP, respectively. These are the areas that would probably be selected if a manual feature selection was performed, based on previous electrophysiology studies which show how ERD is originated contralaterally to the movement [7], while MRCP is generated closer to the midline vertex [8].

### Experiment 2

#### Electrophysiology analysis

**Figure 8:**
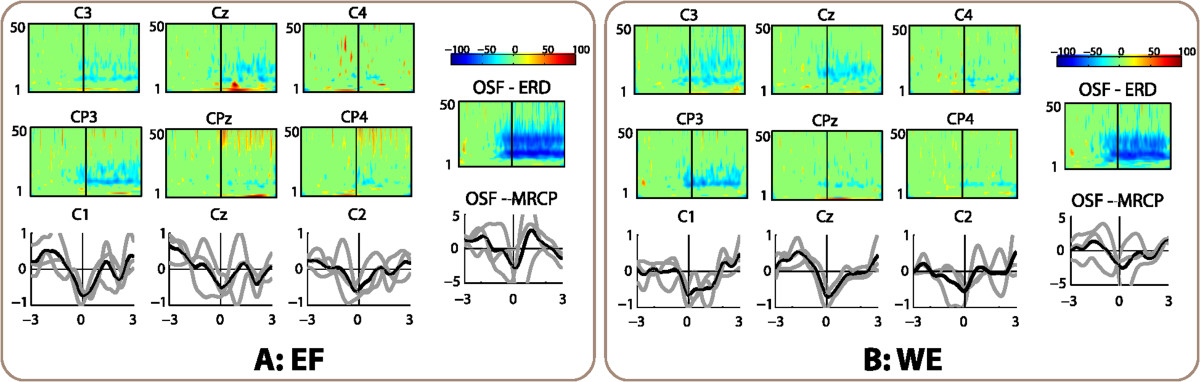
**Electrophysiology analysis of the two movements performed by the patients, averaged for the three of them.** Panels **A-B** correspond to the analysis for each of the two movements that patients performed (EF and WE, respectively). The top and center-left areas of each panel show the significant ERD process in six channels around the motor cortex (*x* axis corresponds to the time interval [-3, 3], *y* axis represents the frequency range [1-50] Hz). Bottom-left area shows the average MRCPs for each patient (gray lines), and the average of the three patients (black lines) in central channels C1, Cz and C2. Top-right depicts the scale of ERD plots. Center-right shows the ERD in the OSF signal. Bottom-right corresponds to the MRCP of the OSF signal.

Significant *α* and *β* ERD, and MRCPs were found for the three patients for both movements. Figure 8 shows both EEG correlates averaged for the three patients. ERD magnitudes and MRCP amplitudes were similar between the healthy subjects and patients. Furthermore, they were also similar between the executed and the attempted movements by the patients group, suggesting that these EEG correlates can also be used for decoding of movement intention of paralyzed joints in this population of patients.

#### Classification results

**Figure 9:**
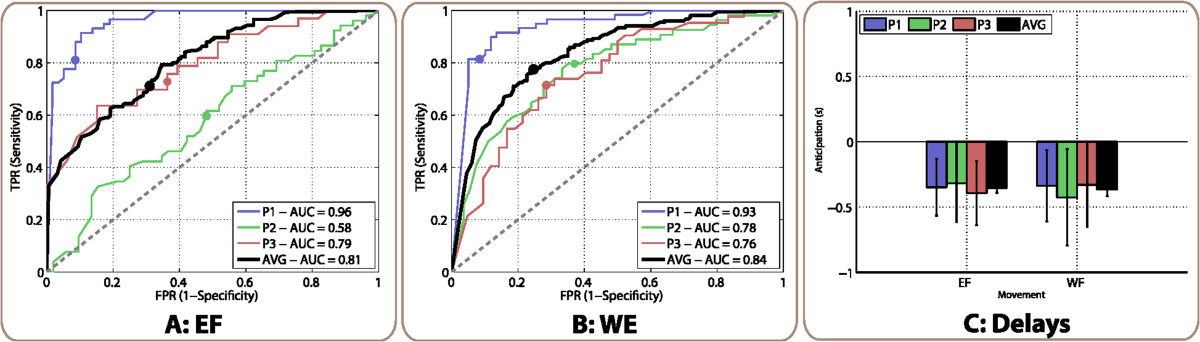
**ROC curves and anticipation analysis for SCI patients.** Panels **(A)** and **(B)** correspond to ROC curves of EF and WE, respectively. Each colored line represents one of the patients, while black lines show the average of the three of them. Circles on each line represent the point of the curve with equal sensitivity and specificity, which are considered the optimal working points. Diagonal gray dashed lines represent the performance of a random classifier. Each legend box contains the AUC for each patient and the average of them. Panel **(C)** shows anticipation analysis for each movement. The *y* axis indicates the anticipation in seconds and the *x* axis the movement type. For each movement, the colored bars indicate the mean ±std anticipation of each patient, and black bars the average anticipation for all of them.

Average ROC curves for movement execution of EF and attempt of WE are shown in Figures 9A-B. The resulting AUC were very similar for both tasks (0.81 for EF and 0.84 for WE), and also similar to the ones obtained for the group of healthy subjects for the same movements (0.86 for EF and 0.77 for WE). For the execution of EF, 71.1*%* of true positives and 31.0*%* of false positives would be obtained with the optimal threshold. For the attempt of WF, the decoder provided 77.5*%* of true positives and 24.7*%* of false positives.

Table 5 shows the percentage of correct trials obtained for the SCI patients. For the execution of EF, 50.2*%* of trials were decoded before the movement onset, while for the attempt of WE the percentage of anticipated trials was 57.7*%*.The anticipation for both tasks was similar, as can be observed in Figure 9C. Average onset detection times were -352 ms for EF and -364 ms for WE.

**Table 5:** **Percentage of correct trials averaged for patients**

	EF	WE	Avg
Avg Patients	50.2±3.8*%*	57.7±5.4*%*	53.9±5.3*%*

## Discussion

The electrophysiology analysis showed that both healthy subjects and the studied SCI patients presented ERD and MRCP activations for the different movements. For the healthy subjects, there were statistical differences dependent on the moving joint for pre-movement *α* ERD. This result complements work by Pfurtscheller et al. [25], which showed statistical differences in post-movement *β* ERS for distal movements (wrist, finger and thumb). In addition to this, we found no statistical differences between movements in MRCP amplitude. These results agree with previous analysis reported in [26], which compared MRCPs for shoulder, elbow and finger movements and only found statistical differences between shoulder and finger. However, as we did not record finger motions, further investigation would be required to study whether there exist variations between MRCP amplitudes in the movements covered in this paper and finger movements.

Decoding results reported for the first experiment show the applicability of BMI on healthy subjects for the three different joints of the arm, with accuracies ranging from 40*%* to 75*%*, dependent on the type of movement. Although studies have shown that the target population of BMI rehabilitation (i.e., paralyzed patients) may have altered brain patterns during motor tasks [15, 46–48], several works have successfully used classifiers based on ERD or in MRCPs to decode motion intention in these paralyzed patients [5, 6, 12–14, 22]. Indeed, results of the second experiment showed similar BMI performances between the healthy group (EF: 60.8*%*, WR: 39.5*%*) and the studied SCI patients (EF: 50.2*%*, WR: 57.7*%*). Despite the fact that performances were higher for elbow than for wrist movements for healthy subjects, this was not the case for SCI patients (which executed elbow movements and attempted wrist movements). On the contrary, accuracy was slightly higher for wrist than for elbow movements for the patients, and also than for wrist movements performed by healthy subjects. As the number of patients in this experiment was limited, further research should be done to verify if significant differences in accuracies between motor execution and motor attempt are present in these patients. This might be explained by the recruitment of additional brain regions during movement attempt when compared to movement execution [49].

Decoding accuracies were dependent on the movement as revealed by the statistical tests. Percentages of correctly anticipated trials ranged from almost 40*%* in the worst movements (wrist) to near 75*%* in the best ones (shoulder). These performances were correlated with both MRCP amplitude and ERD, despite the fact that MRCP amplitudes did not show any statistical difference between movements. A detailed comparison of these results with previous works on continuous decoding of motor intention [10, 12–14, 18–23, 33] is difficult to provide due to differences in the protocols and reported metrics. For instance, in [10] the anticipation window utilized for wrist extension decoding in healthy subjects was larger than the one used in this paper, and the percentage of correct trials they achieved was around 30*%*. In [22] they also considered detections after the movement onset for ankle dorsiflexion, and reported accuracies of about 80*%* with healthy subjects and 55*%* with stroke patients. A recent study with a similar methodology to that presented in this paper decoded around 65*%* of reaching movement trials with healthy and stroke subjects, when considering as valid the onsets detected between -0.75 and 0.75 seconds [33]. The accuracies reported in this paper correspond to trials decoded between -1 second and the movement onset. When also considering the trials decoded up to one second after the movement onset as valid, the average accuracy was increased more than 10*%* (details not reported in this paper).

All the decoding process has been automated, as it has been pointed as an important property for the deployment of BMIs in rehabilitation [50]. For each subject and movement, the proposed decoder was able to select among a large number of features extracted from MRCPs and ERD in a completely automated manner. The combination of both types of features improved significantly the use of each feature alone in terms of correct trials and anticipation, as has also been shown in a parallel work with healthy and stroke subjects [33]. However, as the results presented in this paper were obtained offline with cross-validation, and zero-phase filters were used, a drop in performance might be expected when operating online. Previous works have reported a drop of about 10*%* in true positive rate when changing from offline to online operation in movement decoding using MRCPs [22, 23]. Therefore, further research would be necessary to evaluate the impact of this drop when combining both types of features (ERD and MRCPs) with our optimized feature selection procedure. Interestingly, the automatically selected features were consistent with the electrophysiology analysis and, therefore, could be used as a plausible methodology to induce brain plasticity in neurorehabilitation, or to use natural brain commands for motor substitution.

The EEG decoding results presented in this paper (with anticipated trials ranging from 40*%* to 75*%*) show that the movement chosen for the rehabilitation exercises has a significant impact on the BMI accuracy. These accuracy differences are important in a rehabilitation context as they may have an impact on the applicability of BMI decoders for therapies involving analytic movements. However, it is still unclear how different decoding accuracies can affect the rehabilitation outcome, both in terms of functional recovery or neural reorganization. An interesting pursuit for future research would be to evaluate the relationship between decoding accuracy and rehabilitation outcome, since recent studies that successfully applied online BMI to provide feedback to the subjects did not report performances achieved, but pointed on the importance of the temporal decoding precision [5, 11].

## Conclusions

This work showed the applicability of a BMI on seven upper limb analytic movements (i.e., the seven degrees of freedom of the arm). An experiment with healthy subjects revealed that the seven movements can be decoded before the actual movement onsets, and that there are differences in EEG correlates and decoding performances dependent on the moving joint. A second experiment performed with SCI patients showed how this BMI can be applied in a clinical population. Similar performances were achieved for movements they could and could not perform. Performances were also similar between the patients and the healthy subjects.

## Consent

Written informed consent was obtained from all the healthy subjects and patients for the publication of this report and any accompanying images.

## Authors’ original submitted files for images

Below are the links to the authors’ original submitted files for images. Authors’ original file for figure 1 Authors’ original file for figure 2 Authors’ original file for figure 3 Authors’ original file for figure 4 Authors’ original file for figure 5 Authors’ original file for figure 6 Authors’ original file for figure 7 Authors’ original file for figure 8 Authors’ original file for figure 9
