# Supplementary figures and images for: Continuous decoding of movement intention of upper limb self-initiated analytic movements from pre-movement EEG correlates
Source: J Neuroeng Rehabil. 2014 Nov 15;11:153. doi: 10.1186/1743-0003-11-153 (PMC4247645; doi:10.1186/1743-0003-11-153)

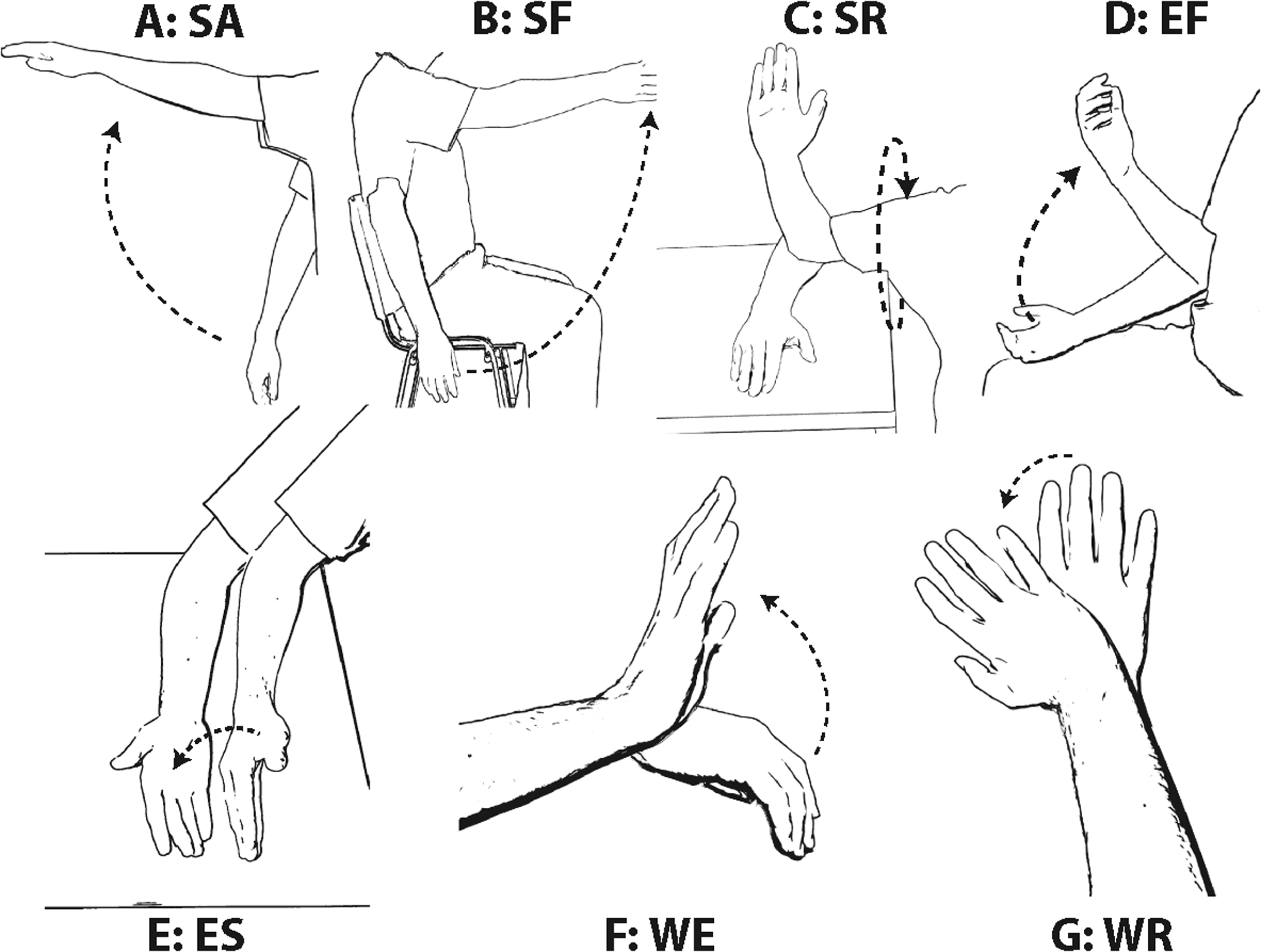

Supplement: Supplementary file 1 — Authors’ original file for figure 1 [file 12984_2014_674_MOESM1_ESM.tif]

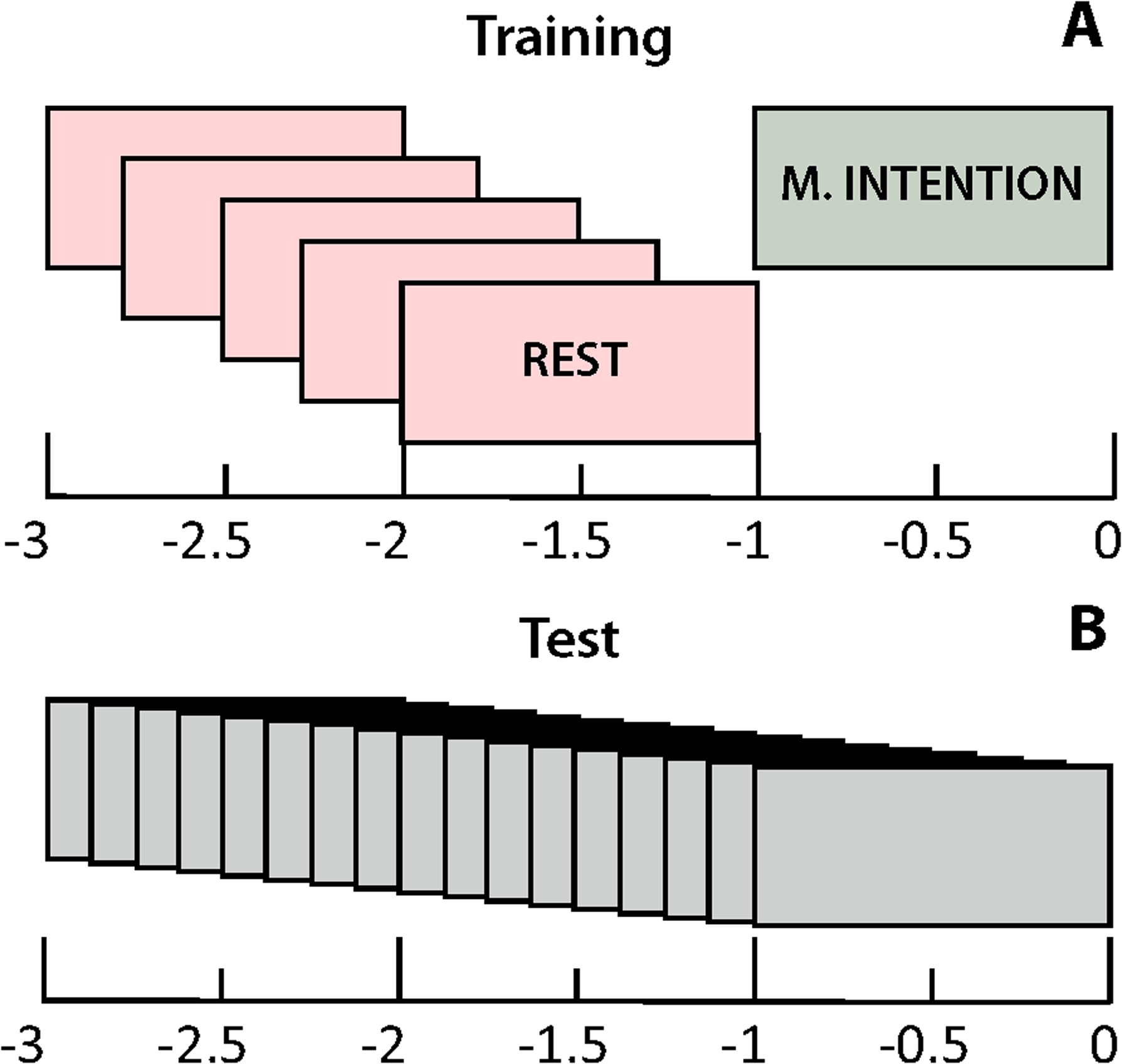

Supplement: Supplementary file 2 — Authors’ original file for figure 2 [file 12984_2014_674_MOESM2_ESM.tif]

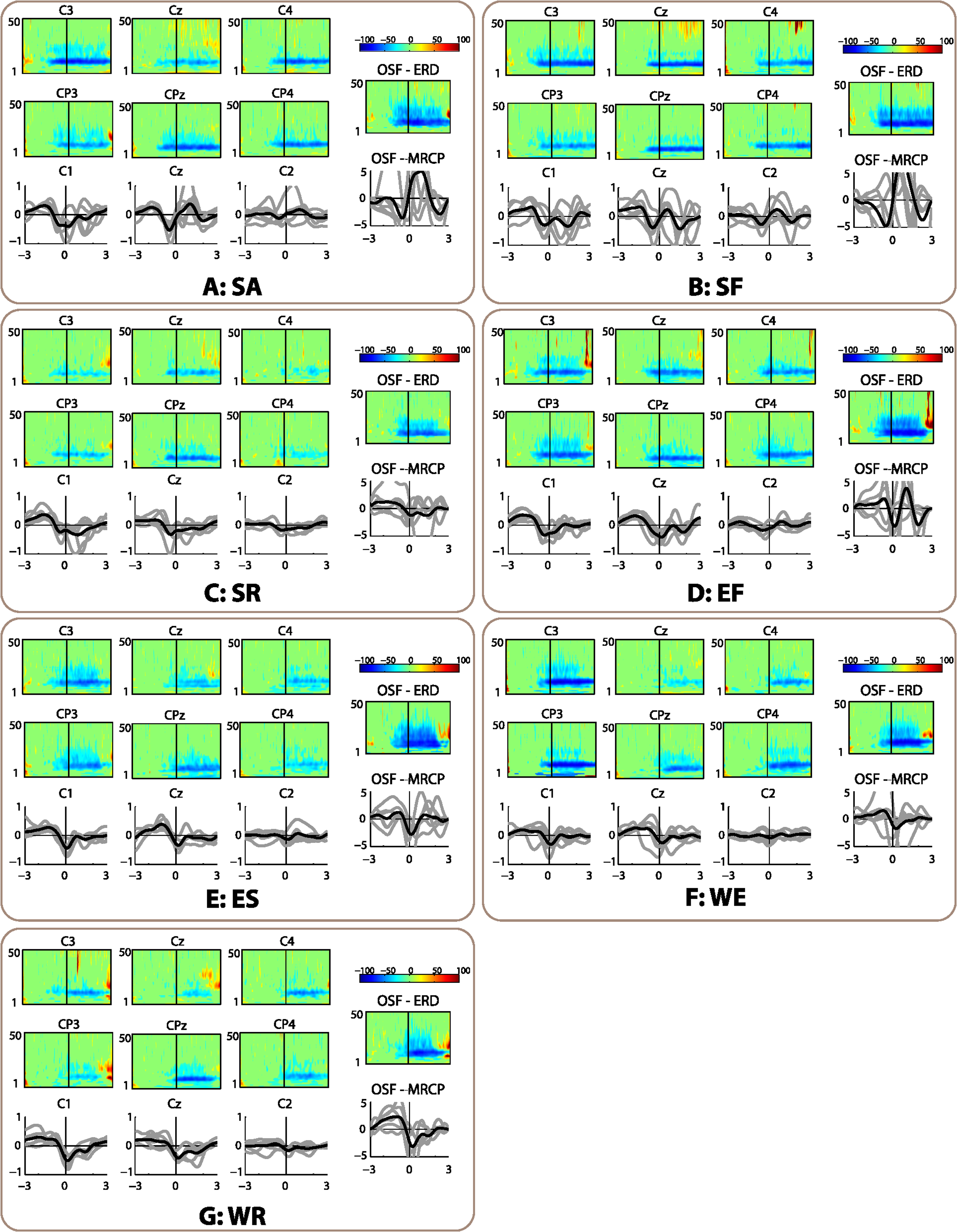

Supplement: Supplementary file 3 — Authors’ original file for figure 3 [file 12984_2014_674_MOESM3_ESM.tif]

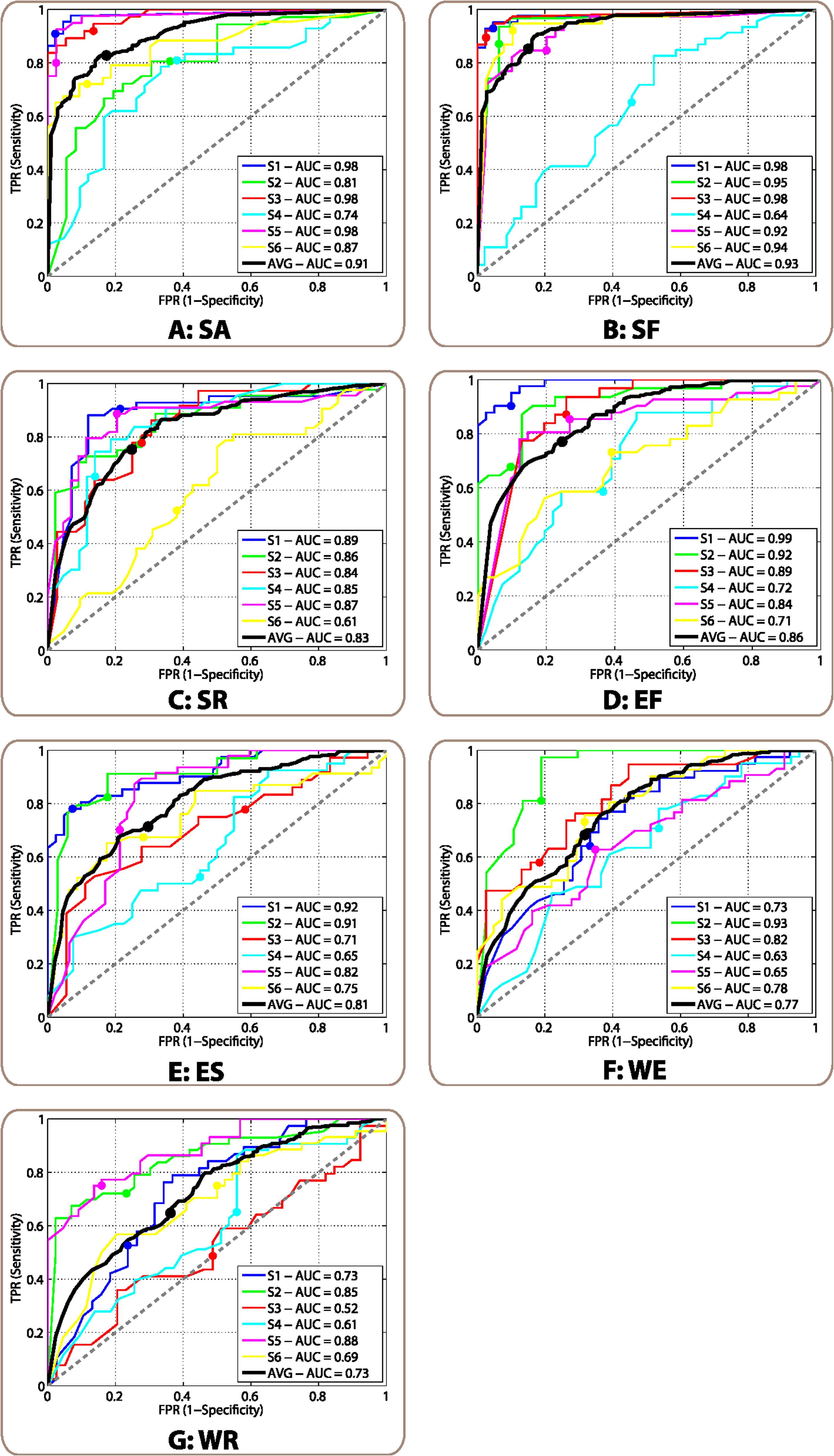

Supplement: Supplementary file 4 — Authors’ original file for figure 4 [file 12984_2014_674_MOESM4_ESM.tif]

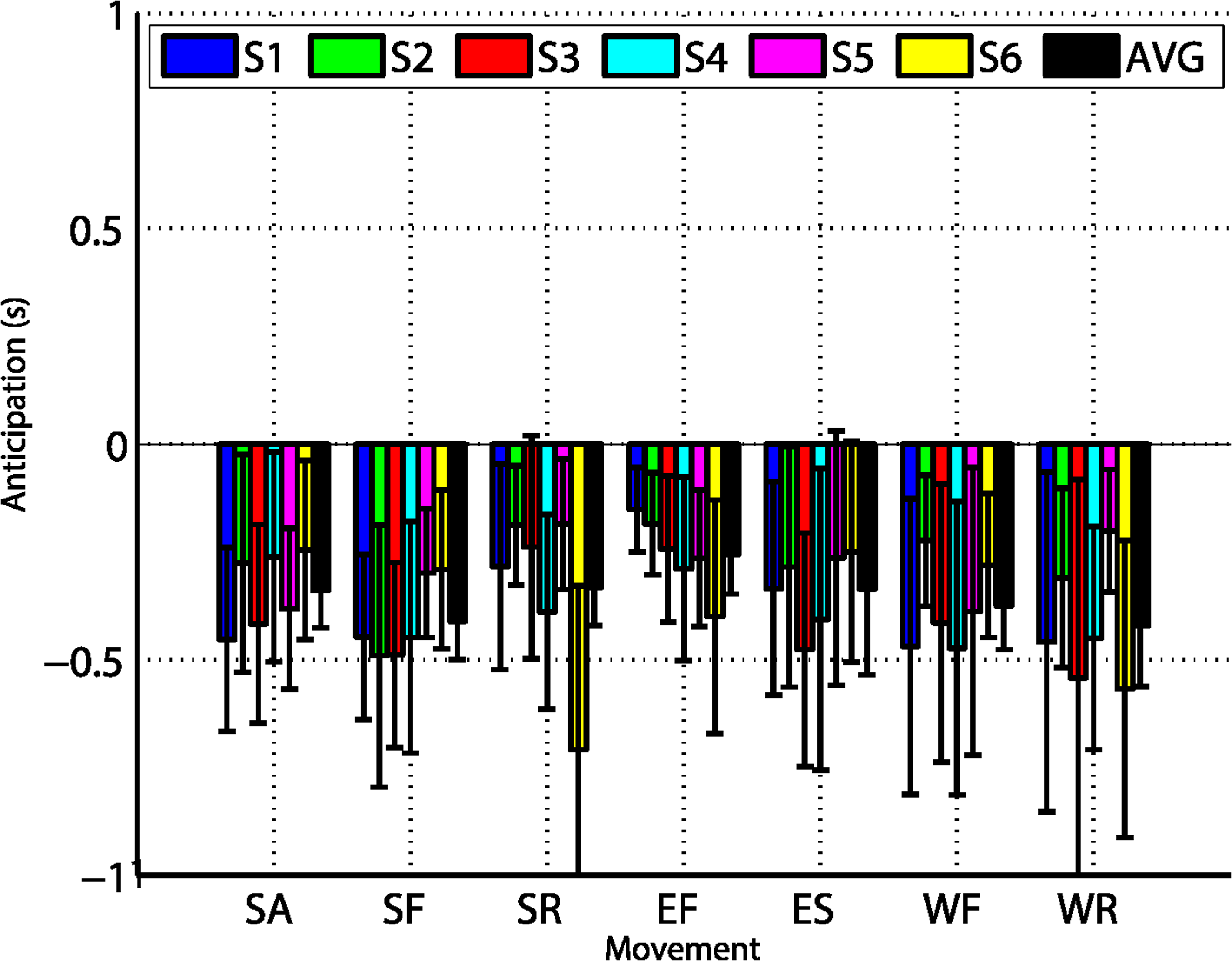

Supplement: Supplementary file 5 — Authors’ original file for figure 5 [file 12984_2014_674_MOESM5_ESM.tif]

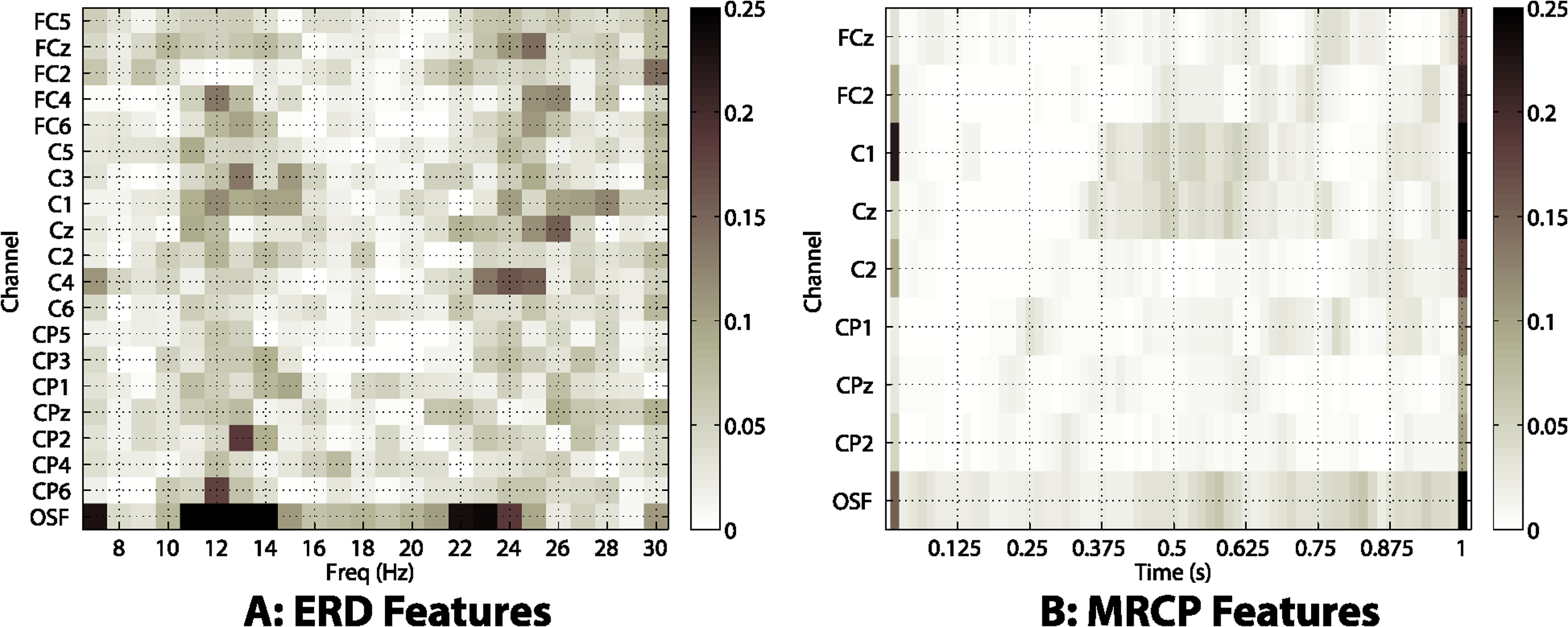

Supplement: Supplementary file 6 — Authors’ original file for figure 6 [file 12984_2014_674_MOESM6_ESM.tif]

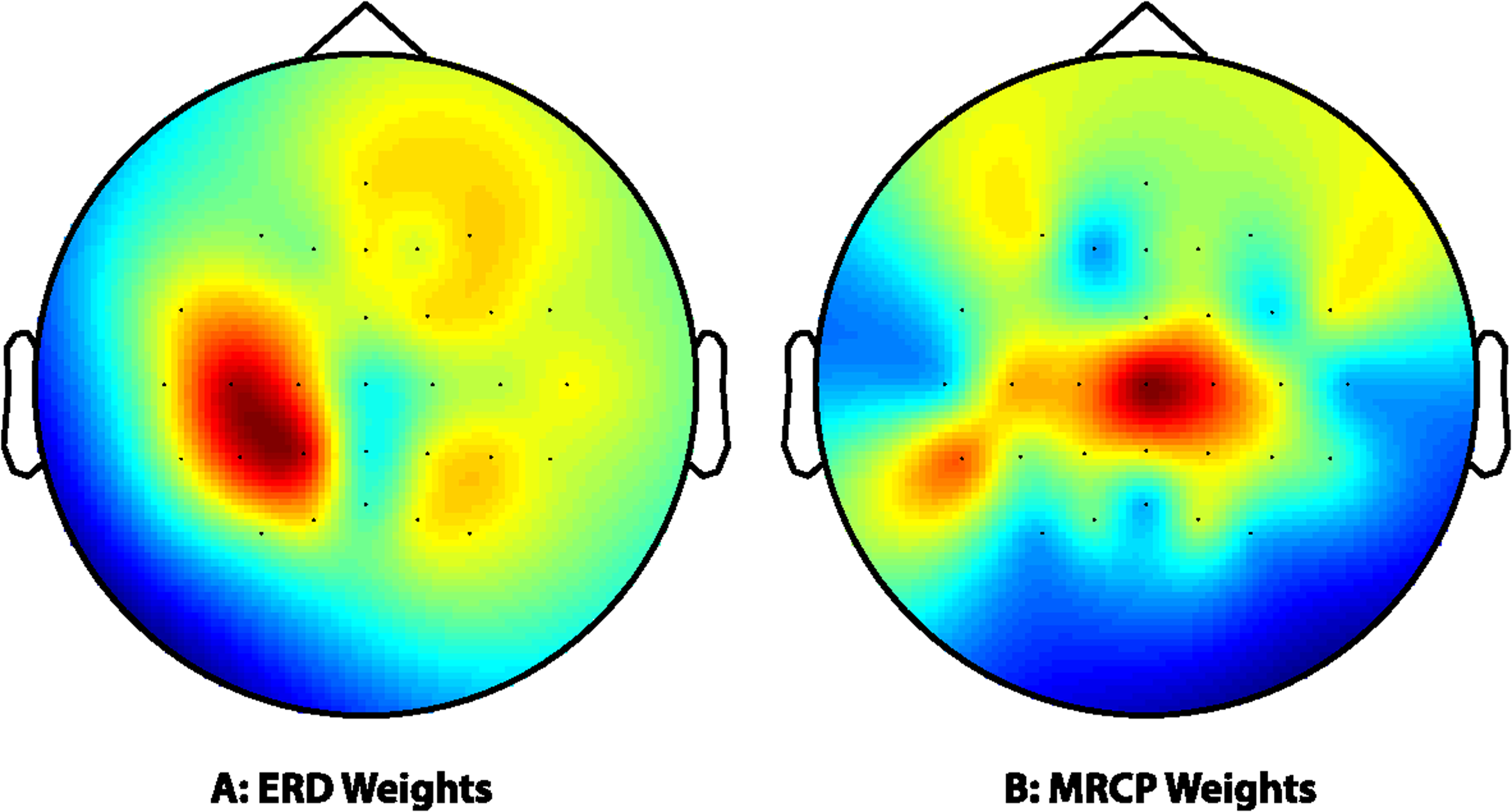

Supplement: Supplementary file 7 — Authors’ original file for figure 7 [file 12984_2014_674_MOESM7_ESM.tif]

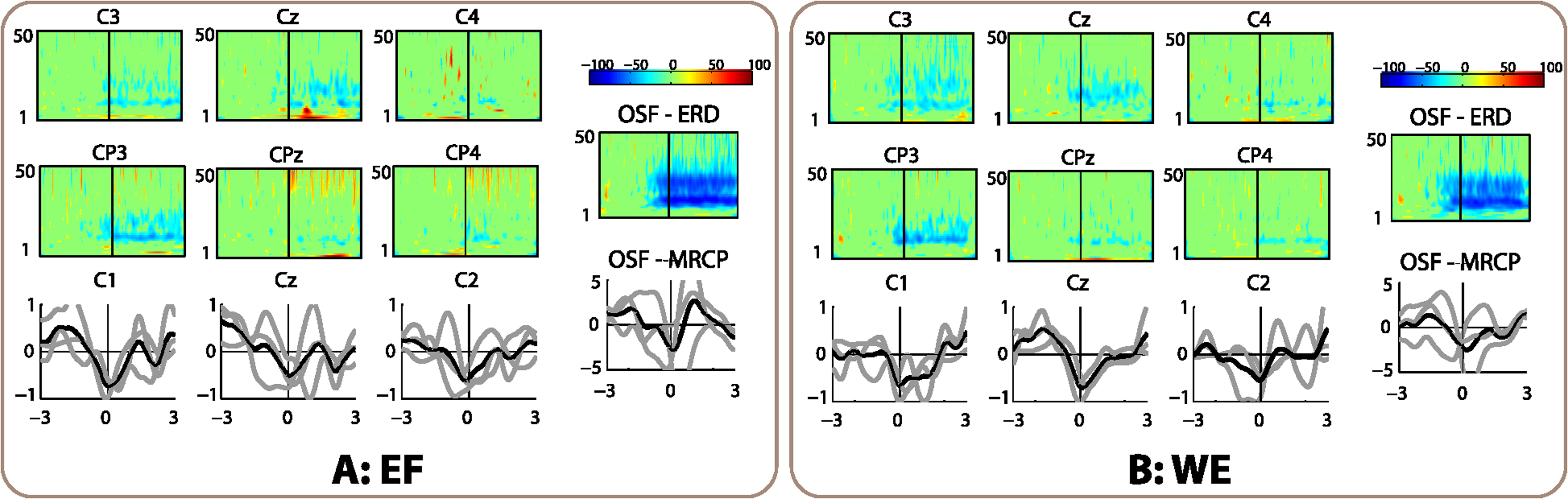

Supplement: Supplementary file 8 — Authors’ original file for figure 8 [file 12984_2014_674_MOESM8_ESM.tif]

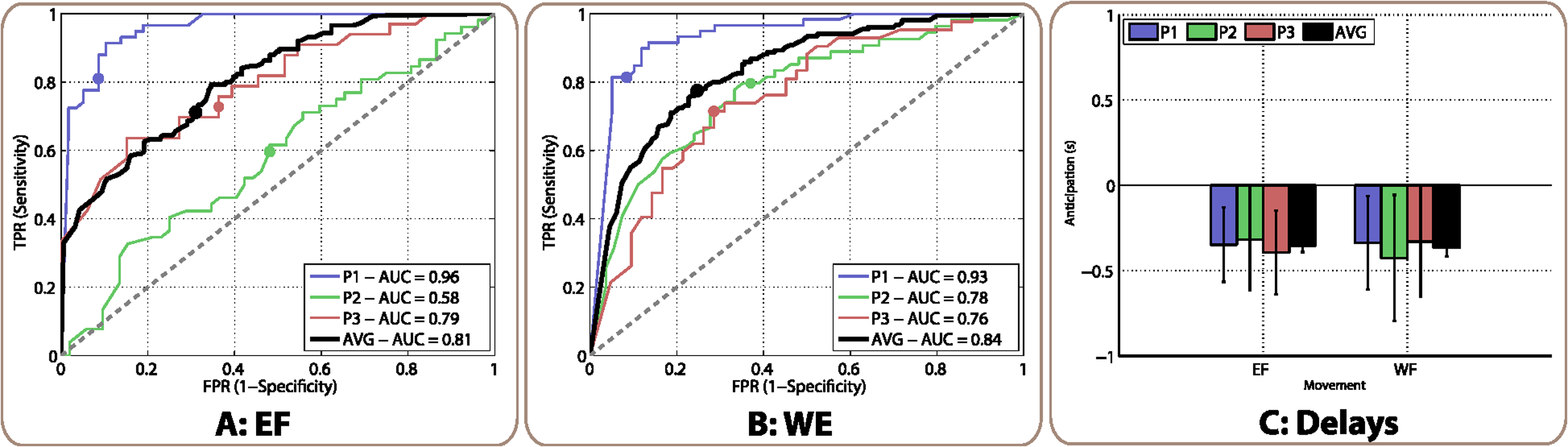

Supplement: Supplementary file 9 — Authors’ original file for figure 9 [file 12984_2014_674_MOESM9_ESM.tif]
